# Exclusion and inequity: a national analysis of disability-inclusive admission criteria in Saudi medical schools

**DOI:** 10.3389/fmed.2025.1667625

**Published:** 2025-09-23

**Authors:** Anwar A. Sayed

**Affiliations:** ^1^Department of Basic Medical Sciences, College of Medicine, Taibah University, Madinah, Saudi Arabia; ^2^King Salman Center for Disability Research, Riyadh, Saudi Arabia

**Keywords:** admission criteria, disability inclusion, equity in medical education, healthcare workforce diversity, inclusive education policies, MBBS programs, medical school admissions, Saudi Arabia

## Abstract

**Introduction:**

Equitable access to medical education is essential for building a diverse and inclusive healthcare workforce. Despite national disability legislation in Saudi Arabia, the extent to which undergraduate medical programs implement inclusive admission criteria remains unclear. This study evaluates the inclusiveness of admission policies for students with disabilities across all undergraduate MBBS programs in Saudi Arabia.

**Methods:**

A cross-sectional review of the official admission policies of 32 universities was conducted. Data were extracted from university websites to identify whether institutions provided inclusive pathways, accommodations for disability, or used exclusionary language or environmental/institutional barriers related to disability.

**Results:**

Only 6 of 32 universities–exclusively public institutions–had formal disability-inclusive policies. The remaining schools either lacked inclusive provisions, support structures or required medical fitness documentation that could exclude applicants with physical, sensory, or mental impairments. Private universities showed no evidence of inclusive admissions.

**Discussion:**

Most Saudi medical schools maintain exclusionary, ambiguous, or unsupportive admission practices, undermining national commitments to disability rights and global Sustainable Development Goals (SDGs 4 and 10). Urgent institutional reforms are needed to dismantle barriers and ensure transparency, equity, supportive environments, and accessibility for applicants with disabilities in health professions education.

## 1 Introduction

Inclusive education is a fundamental principle in higher education, ensuring that all qualified individuals have access to learning opportunities regardless of their physical abilities. Inclusivity in admissions into undergraduate medical programs (MBBS) has become particularly critical. This is because a diverse physician workforce can contribute to improved patient care, better representation of marginalized communities, and a more holistic approach to healthcare delivery (1).

Globally, medical education institutions have been adapting their admission policies to accommodate students with disabilities, recognizing their potential contributions to the medical profession (2). However, despite these efforts, significant barriers remain, particularly in the selection process for medical school applicants.

The commitment to inclusive and equitable medical education is deeply aligned with global initiatives such as the United Nations Sustainable Development Goals (SDGs). Specifically, SDG 4 aims to “ensure inclusive and equitable quality education and promote lifelong learning opportunities for all,” while SDG 10 calls for reducing inequalities within and among countries (3). In the context of medical education, ensuring equitable access for students with disabilities is not only a matter of national policy compliance but also an ethical and global imperative. As healthcare systems increasingly recognize the value of diversity in the workforce, attention must be paid to dismantling systemic barriers that prevent underrepresented groups, including individuals with disabilities, from entering and thriving within medical professions.

Medical schools often set rigorous admission criteria that include cognitive, psychomotor, and physical competencies. While such requirements are necessary to ensure the capability of future physicians, they may unintentionally exclude individuals with disabilities (4, 5) who, with appropriate accommodations, could successfully complete medical training. Many countries have implemented policies to promote inclusivity, offering reasonable adjustments in admission tests, curricula, and clinical training. However, admission criteria remain restrictive in some regions, with limited flexibility in accommodating disabled students (6).

In Saudi Arabia, MBBS programs are among the most competitive, with stringent selection processes to ensure high academic and professional standards. While national education and disability policies advocate for equal opportunities, it is unclear to what extent these principles are reflected in medical school admissions. The Kingdom has demonstrated a strong commitment to supporting individuals with disabilities through its participation in the “Convention on the Rights of Persons with Disabilities” and its “Vision 2030” initiative, which prioritizes the empowerment and full inclusion of people with disabilities in all aspects of society (7). However, despite these national efforts, the extent to which medical school admission policies align with these commitments remains uncertain.

The Law of Disability in Saudi Arabia (2000) provides a foundational definition, characterizing a person with disability as one experiencing a permanent partial or total deficiency in capabilities, resulting from an impairment that hinders meeting the normal requirements of life within society (8). This broad definition encompasses physical, sensory, intellectual, and psychosocial impairments and mandates equal opportunities in education. However, a discernible gap exists between this overarching legal mandate and its specific application within the stringent, competency-based admission frameworks of medical schools. The operationalization of inclusivity often hinges on institutional interpretation of functional capacity and reasonable accommodation, areas where policy may remain ambiguous or exclusionary (5, 9).

This study aims to systematically examine the admission criteria for MBBS programs across medical schools in Saudi Arabia and evaluate their inclusivity toward applicants with disabilities. By analyzing institutional policies, this research seeks to determine whether current admission requirements support or hinder access for disabled students. The findings will provide insights into potential policy gaps and contribute to ongoing discussions about the need for inclusive and equitable medical school admissions in Saudi Arabia.

## 2 Materials and methods

### 2.1 Study design and data collection

This study was a descriptive, cross-sectional examination aimed at investigating the admission criteria and policies for students with disabilities in medical colleges across Saudi Arabia. All information was retrieved from its original sources, i.e., official university websites.

Admission criteria were retrieved from the College of Medicine of each university. In case there were no specific admission criteria for the undergraduate MBBS program, the general admission criteria of the university were retrieved. The aim of examining the admission criteria was to determine whether these institutes have a formalized admission policy for students with disabilities. Moreover, if present, the offered accommodations for their admission were recorded.

The order in which the included universities are present is based on their rank according to the Saudi Commission for Health Specialties’ latest ranking of students’ performance in the Saudi Medical Licensure Exam (SMLE) (10). The data collection took place between August 2024 and January 2025.

The tuition fees were also collected in Saudi Arabia’s national currency, i.e., Saudi Riyals. The fees were also presented in the US dollars through the following fixed currency exchange rate: 1 USD = 3.75 SAR (11).

### 2.2 Study setting

The study was conducted across all recognized medical colleges in Saudi Arabia. These institutions were identified using an official list obtained from the Ministry of Education and the Saudi Commission for Health Specialties. The inclusion criteria of this study are universities in Saudi Arabia that are either public or private and have an established undergraduate MBBS program recognized by the Saudi Commission for Health Specialties. Excluded universities were those without an MBBS program at the time of the data collection.

### 2.3 Data analysis

This analysis of the data collected through this study included document analysis, in which admission criteria, either general to the university or specific to the MBBS programs, were carefully examined. These criteria were analyzed to determine if it has any exclusionary criteria, such as “Students must be medically fit” or “Students should be medically cleared”. Such statements may require students to submit a form filled out by a physician declaring that students are free of physical or mental disabilities.

A step-wise approach has been adopted to analyze the data of this study (12). Descriptive statistics were used to describe the data collected throughout the study. Frequencies and percentages were used to describe categorical data, whereas the distribution of numerical data was determined using the Shapiro Wilk test. Parametric data was described using mean (±standard deviation), whereas non-parametric data was described using median and interquartile ranges.

The association between the nature included universities, locations, and their exclusionary admission criteria was assessed using the Chi-square test.

### 2.4 Ethical considerations

This study did not recruit or involve any human participants. Additionally, no sensitive and private information were collected as part of this study. Hence, given the nature of this study, the study did not require an institutional review board (IRB) ethical approval.

## 3 Results

### 3.1 University characteristics

**FIGURE 1:**
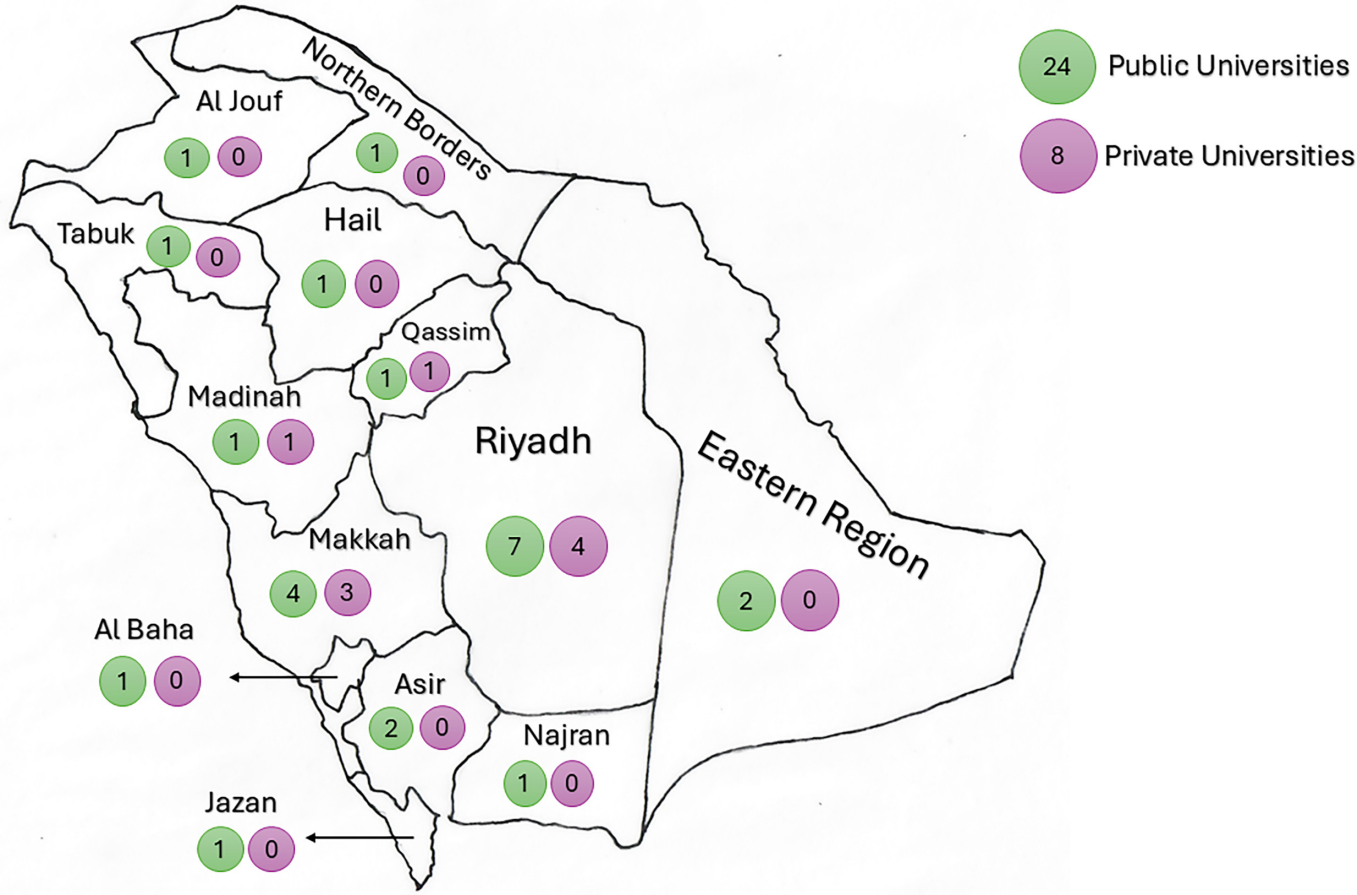
The universities’ distribution across Saudi Arabia. The figure demonstrates a map of Saudi Arabia, showing the distribution of public (green circles) and private (purple circles) universities in every administrative region. The number inside each circle represents the number of universities of each type (public or private).

A total of 32 universities were included in this study, distributed across the 13 administrative regions of Saudi Arabia. The majority of these universities (*n* = 24) were public universities, whereas 8 of them were private universities. The region of Riyadh, where the capital city of the country is located, has the highest number of universities (*n* = 11) that deliver undergraduate MBBS programs, followed by the region of Makkah (*n* = 7). Each of the Eastern and Asir regions has two public universities, while the remaining regions have at least one public university and may have a private one. The distribution of the universities across the regions is detailed in Figure 1.

Public universities do not require tuition fees for the undergraduate MBBS programs. On the other hand, private universities require tuition fees for undergraduate MBBS programs. The mean annual tuition fees are SAR 79,875 ± 12,076 (equivalent to $21,300 ± 3220.27). Almost all (7 out of 8) of the private universities provide forms of discounts to their students, primarily for excellent students, e.g., annual GPA of over 90% or 95%. Some of these universities (*n* = 3) facilitate the payments of their tuition fees through their acceptance of payments in installments.

### 3.2 MBBS programs establishment and national performance

The first university to deliver the MBBS program was the public King Saud University in the capital city of Riyadh in 1967. Subsequently, public universities continued to be established, which also delivered MBBS programs, with the latest to be established being Princess Nourah Bint Abdulrahman University and the University of Bisha, being established in 2013. The concept of private universities which delivered undergraduate MBBS programs started in 2004 with the establishment of Ibn Sina National College for Medical Studies in Jeddah (Makkah region). The private university Al-Rayyan Colleges of Health Sciences and Nursing in Madinah (Madinah region) was the latest private university to be established, which is less than 10 years old, and was founded in 2017.

The Saudi Commission for Health Specialties, the regulatory body of medical practice in Saudi Arabia, has ranked universities based on their students’ performance in the Saudi Medical Licensure Exam (SMLE) in the last 3 years. The Commission then grouped universities into five categories: Group A (*n* = 3): Universities that showed excellence in their performance; Group B (*n* = 7): Universities with performance above the national average; Group C (*n* = 11): Universities with average national performance; Group D (*n* = 6): Universities with less than average national performance, with room for improvement in certain specialties; Group E (*n* = 5): Universities with the lowest national performance. The establishment of the medical colleges that delivered the MBBS programs and the universities’ performance categorization is detailed in Table 1.

**TABLE 1:** List of Universities that deliver MBBS programs in Saudi Arabia.

University name	Rank	Category	Region	City	Inclusive admissions	CoM establishment
Taibah University	1	A	Madinah	Madinah	Yes	1998
King Saud University	2	A	Riyadh	Riyadh	Yes	1967
King Saud Bin Abdulaziz University For Health Sciences	3	A	Riyadh	Riyadh	No	2005
Sulaiman Alrajhi University	4	B	Qassim	Al Bukayriyah	No	2009
Qassim University	5	B	Qassim	Al meledaa	No	2000
Imam Abdulrahman Bin Faisal University	6	B	Eastern province	Dammam	No	1975
Alfaisal University	7	B	Riyadh	Riyadh	No	2008
King Abdulaziz University	8	B	Makkah	Jeddah	Yes	1975
Majmaah University	9	B	Riyadh	Al Majma’ah	No	2009
Princess Nourah Bint Abdulrahman University	10	B	Riyadh	Riyadh	No	2013
Jouf University	11	C	Al jouf	Sakaka	No	2005
University of Tabuk	12	C	Tabuk	Tabuk	No	2006
Taif University	13	C	Makkah	Taif	Yes	2007
Najran University	14	C	Najran	Najran	Yes	2010
University of Hail	15	C	Hail	Hail	No	2005
Imam Mohammad Ibn Saud Islamic University	16	C	Riyadh	Riyadh	No	2008
Jazan University	17	C	Jazan	Jizan	Yes	2006
Jeddah University	18	C	Makkah	Jeddah	No	2010
Umm Al-Qura University	19	C	Makkah	Makkah	No	1996
King Faisal University	20	C	Eastern province	Hofuf	No	2001
King Khalid University	21	C	Asir	Abha	No	1980
Al-Rayyan Private Colleges of Health Sciences and Nursing	22	D	Madinah	Madinah	No	2017
Prince Sattam Bin Abdulaziz University	23	D	Riyadh	Al kharj	No	2008
Batterjee Medical College	24	D	Makkah	Jeddah	No	2005
Shaqra University	25	D	Riyadh	Shaqra	No	2011
University of Bisha	26	D	Asir	Bisha	No	2013
Northern Borders University	27	D	Northern Borders	Arar	No	2007
Al-Baha University	28	E	Al baha	Al baha	No	2008
Almaarefa University	29	E	Riyadh	Diriyah	No	2009
Dar AL Uloom University	30	E	Riyadh	Riyadh	No	2012
Ibn Sina National College for Medical Studies	31	E	Makkah	Jeddah	No	2004
Vision Colleges	32	E	Riyadh-Makkah	Riyadh-Jeddah	No	2009

CoM, College of Medicine.

### 3.3 The inclusivity and exclusionary of the admission criteria into MBBS programs

All of the included universities displayed admission criteria on their respective websites. However, some of these universities did not specify admission criteria for their MBBS programs. Only six universities (out of 32) stated the admission criteria for students with disability. These were Taibah University, King Saud University, King Abdulaziz University, Taif University, Najran University, and Jazan University. Interestingly, all these universities are public, either with a dedicated admission portal for students with a disability or a dedicated preparatory track they can be admitted to before joining the MBBS program.

**FIGURE 2:**
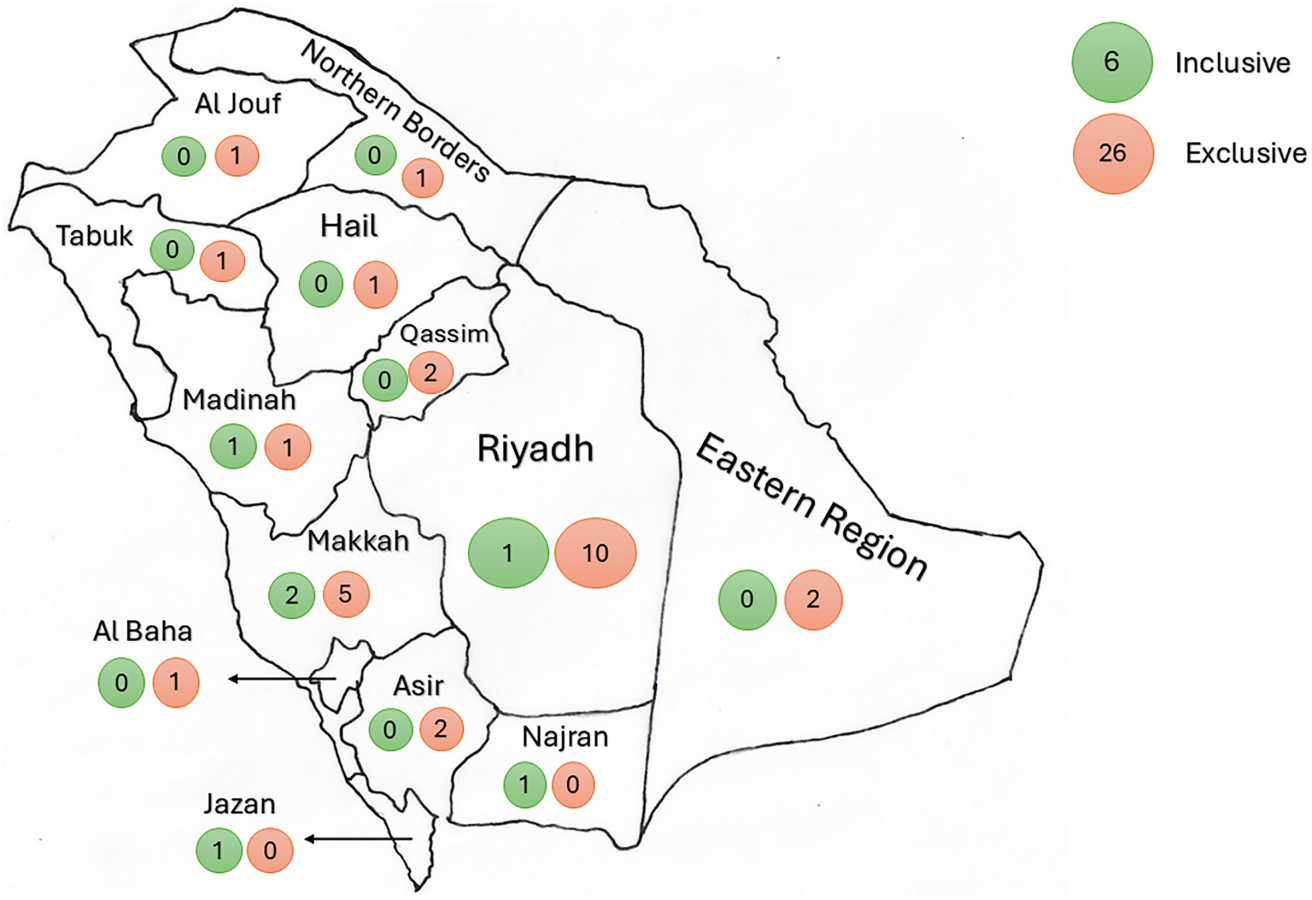
The inclusive and exclusive universities’ distribution across Saudi Arabia. The figure demonstrates a map of Saudi Arabia, showing the distribution of universities with inclusive admission criteria (green circles) and those exclusive universities (red circles) in every administrative region. The number inside each circle represents the number of universities of each type (inclusive or exclusive).

The remaining universities demonstrated direct and indirect exclusionary admission criteria into MBBS programs. Direct exclusionary admission criteria have direct statements that students should be “medically fit” or “clear of any form of disability,” such as the case at Qassim University. As part of their admission requirements, prospective students are required to upload a Medical Fitness Form signed by a qualified practicing physician. This form indicates that the student is free from hearing difficulties, wearing glasses, mental disorders, mobility impairment, or physical disability. Universities that were indirectly exclusive toward students with disability did not indicate any special measures or admission criteria for students with disability. Furthermore, the absence of any mention of disability accommodations or support services within the admission criteria or related university policy pages was interpreted as potential indirect exclusion. This could manifest in practice as a lack of accessible facilities (e.g., clinics, simulation labs), inflexible curriculum delivery methods that do not accommodate diverse learning needs, insufficient provision of assistive technologies, or the absence of dedicated disability support offices – all creating *de facto* barriers even without explicit exclusionary language in admission requirements. Interestingly, none of the private universities displayed any inclusive admission criteria for students with disability. The distribution of inclusive universities across the country is demonstrated in Figure 2.

The association between the type of university, whether public or private and their inclusive or exclusionary admission criteria was assessed (Table 2).

**TABLE 2:** Association between the universities’ types and their admission criteria.

University type	Admission criteria		*P*-value
	Inclusive	Exclusive	
Public Universities	6	18	0.058
Private Universities	0	8	

Although all the inclusive universities were public, the association between their type and admission criteria did not reach statistical significance (*P*-value = 0.058).

## 4 Discussion

This study provides a comprehensive overview of the admission criteria for students with disabilities into undergraduate MBBS programs across Saudi Arabia. The findings of this study indicate that while some institutions have formalized policies and accommodations, the majority of universities that deliver MBBS programs either lack clear admission criteria for students with a disability or have admission criteria that are restrictive to students with disability.

In Saudi Arabia, admission into medical schools requires a high score calculated from students’ performance in high school (40%), the national aptitude test (30%), and the national comprehensive scientific test (30%). With these calculations in mind, MBBS programs are the most competitive nationally. Currently, some universities do not clearly state any considerations or admission criteria for applicants with a disability. Hence, without dedicated seats, quota in medical school for students with disability, or a dedicated admission process, applicants with disability are unlikely to secure admission compared to high-achieving students (13). Basing the entire selection process on exam results is not entirely accurate and does not predict academic success in medical school (14, 15).

Unfortunately, the restrictive nature of admission criteria in Saudi universities is not limited to undergraduate MBBS programs. According to the findings of this study, over 80% of the universities in Saudi Arabia, regardless of whether they are public or private, display restrictive admission criteria into MBBS programs. Such a finding aligns with and significantly extends the earlier finding by Madhesh, who described the exclusionary admission policies for applicants with disability focusing solely on public universities (16). While Madhesh concluded that the majority of public universities had restrictive policies developed by “body-abled” academics, the present study reveals that this exclusionary trend is even more pervasive, encompassing the vast majority of both public (18/24) and all private (8/8) institutions. This comprehensive national analysis underscores that the problem identified by Madhesh within the public sector is endemic across the entire Saudi medical education landscape, highlighting an even more urgent need for systemic reform (16, 17).

The findings of this study directly contribute to the advancement of SDGs 4 and 10 by highlighting the persistent structural barriers that inhibit equitable access to medical education for students with disabilities in Saudi Arabia (3). Saudi Arabia introduced “The law of disability in Saudi Arabia” in 2000, stating their rights and protecting their welfare (8). Although 26 colleges of medicine (out of 32) were established after the enactment of the disability law in 2000, only six have implemented formal inclusive admission processes. Among these, three universities (Taif University, Najran University, and Jazan University) were established after the law came into effect. This finding indicates that the date of medical college establishment was not a decisive factor in the adoption of inclusive policies. For example, both medical colleges at King Saud University and Taibah University were established prior to this law’s introduction, yet both were inclusive of applicants with disabilities. The rigorous nature of medical training and practice necessitates careful consideration of technical standards required for safe and effective patient care. A critical concern often raised is the ability of students with disabilities to meet the demands of national licensure examinations and subsequent clinical practice. Evidence from other contexts, such as the United States, suggests that medical students with disabilities, particularly physical or sensory disabilities who receive appropriate reasonable accommodations, perform comparably on licensing exams like the USMLE Step 1 to their non-disabled peers (9, 15). While specific data on performance in the Saudi Medical Licensure Exam (SMLE) by students with disabilities is currently lacking, the principle remains that accommodations tailored to individual needs (e.g., extended time, assistive technology, modified simulation equipment) are crucial for enabling equitable demonstration of competency (15, 18). It is essential to differentiate between the core competencies required for safe medical practice and non-essential technical standards that may inadvertently exclude capable individuals; accommodations address the latter without compromising the former (18, 19).

Beyond licensure, the professional trajectory and ethical dimensions of physicians with disabilities warrant attention. Research indicates that doctors with disabilities can perform effectively across various specialties, often bringing unique strengths such as enhanced empathy, improved communication with patients experiencing disability, and diverse problem-solving perspectives, thereby enriching patient care and the healthcare workforce (19, 20). However, they frequently encounter significant ethical challenges, including workplace discrimination, stigma, lack of adequate support, and even harassment, which contribute to higher rates of burnout and attrition (20). These challenges represent a failure of the healthcare system to uphold its ethical obligations toward its own professionals and undermine efforts to build a diverse workforce. Medical schools, therefore, have a dual ethical responsibility: firstly, to ensure equitable access through inclusive admissions and robust support systems, preparing students with disabilities for success; and secondly, to actively combat stigma and ableism within the learning environment and the broader profession, fostering a culture of inclusion that extends into postgraduate training and practice (17, 19, 20).

The findings of this study are also in line with findings elsewhere despite the presence of national disability laws. For example, the exclusionary admission policies described in this study have also been reported in the United States, India, and Brazil (20). In a nationwide study in the United States, Zazove and colleagues examined the admission criteria of 173 accredited medical schools, of which 93% of them included restrictive technical standards (9). Furthermore, these institutes lacked transparency and used vague language that was more likely to discourage applicants with a disability. Noticeably, these findings were reported after the introduction of the Americans with Disabilities Act in 1990, which mandated all educational establishments to avail accommodations for students with disabilities (2). Similarly in India, Singe reported that the Medical Council of India has issued admission guidelines for applicants with disability. Such guidelines were issued in response to India’s Rights of Persons with Disabilities Act. However, these guidelines are unfair, arbitrarily restrictive toward applicants with disability, and in direct violation of the law (21).

Several points of strength could be appreciated in this study. This study is the first of its kind in Saudi Arabia to assess the admission criteria into medical schools across the country. The bilingual examination of all documentation, i.e., Arabic and English policies, allowed for a thorough examination of these documents and reaching accurate conclusions. Moreover, this study included both public and private universities in its analysis, which provided a more comprehensive examination of the current situation in Saudi Arabia.

On the other hand, several limitations could be observed in this study. This study relied entirely on its data collection and analysis of the documented admission policies in the booklet or on the universities’ websites. This exclusive reliance on publicly available documents constitutes the primary limitation. Such reliance may lead to inaccurate results as some of these booklets/websites may be outdated. Crucially, the presence or absence of written policies, even if accurately presented online, does not necessarily equate to their consistent implementation or reflect the actual lived experience of applicants with disabilities. Policies might exist on paper but be poorly communicated, inconsistently applied by admissions staff, or undermined by inaccessible processes or environments not detailed in the admission criteria themselves. Conversely, some universities lacking formal written inclusive policies might offer *ad hoc* accommodations in practice, though this seems unlikely given the overall findings. This gap between documented policy and operational reality limits the ability to fully ascertain the practical inclusivity of the admissions process at each institution and affects the generalizability of our findings regarding the actual barriers faced by applicants. Additionally, the reliance on written policies, even if they were presented accurately on their websites, does not necessarily reflect the reality of the admission process. Interviewing admission officers may have confirmed and strengthened this study’s findings by providing further insights into the admission process and clarifying the supportive measures, if present, for applicants with a disability.

Future studies would benefit significantly from the inclusion of admission officers and students with disability who were accepted into undergraduate MBBS programs. Specifically, rigorous qualitative research employing interviews or surveys with admissions officers, faculty members, disability service providers, and importantly, students with disabilities (both successful applicants and those potentially deterred from applying) is essential. Such research would provide invaluable insights into: (1) the interpretation and implementation of admission policies in practice, including reasons for discrepancies between written policy and action; (2) the nature and adequacy of support services provided throughout the application process and the medical program; (3) the perceived barriers and facilitators encountered by students with disabilities; and (4) the attitudes and awareness levels of key stakeholders regarding disability inclusion in medical education. This deeper understanding is critical for designing effective, context-specific interventions to dismantle barriers and foster genuine inclusion.

## 5 Conclusion

This study reveals substantial gaps in the inclusivity of admission policies for students with disabilities across Saudi medical schools, highlighting a pressing need for policy revision and systemic reform. Despite the existence of national disability rights legislation and international commitments to equity, most institutions maintain restrictive or opaque admissions processes that hinder the participation of students with disabilities in medical education. These exclusionary practices run counter to the principles of social justice and global efforts such as the SDGs. To foster a truly inclusive learning environment, medical schools should implement transparent, accessible, and supportive admission pathways, supported by faculty development and institutional accountability. Engaging stakeholders, including students with disabilities and admissions personnel, in the reform process will be essential to ensuring equitable access and meaningful participation in the healthcare workforce of the future.

## Data Availability

The original contributions presented in this study are included in this article/supplementary material, further inquiries can be directed to the corresponding author.
